# A Rare Case of Intraductal Papilloma Arising from Minor Salivary Gland in the Floor of the Mouth

**DOI:** 10.1155/2020/8882871

**Published:** 2020-08-25

**Authors:** Agnes Assao, Silas Antonio Juvencio de Freitas Filho, Luiz Antônio Simonetti Júnior, Denise Tostes Oliveira

**Affiliations:** ^1^Department of Surgery, Stomatology, Pathology and Radiology (Area of Pathology), Bauru School of Dentistry, University of São Paulo, Bauru, São Paulo, Brazil; ^2^Private Practice, Bauru, SP, Brazil

## Abstract

A 77-year-old woman with a rare oral intraductal papilloma arising from the minor salivary gland located on the floor of the mouth and causing the mucus retention is reported. Microscopically, the lesion was characterized by unicystic cavity exhibiting the lumen partially filled by papillary projections of the ductal epithelium with varying degree of oncocytic metaplasia. Based on the histopathological analysis, the differential diagnosis of oral intraductal papillomas and other ductal neoplasms of salivary origin are discussed.

## 1. Introduction

The incidence of oral tumors arising from the salivary ductal glands, such as intraductal papillomas, is difficult to determine because different terminology has been used for the denomination of the same lesion [1]. Specifically, oral intraductal papilloma in minor salivary glands is a benign solitary tumor characterized by intracystic papillary growth and duct-like structures that affect predominantly the lip and buccal mucosa of the 50-year-old or older patients [1–6]. The occurrence of intraductal papilloma on the floor of the mouth is extremely rare, and there are only a few cases reported in the English literature [4, 5].

We reported a rare intraductal papilloma that developed in the sublingual region involving a minor salivary duct gland and the differential diagnosis of this lesion with other ductal neoplasms of salivary origin are discussed.

## 2. Case Report

A 77-year-old woman with a two months history of swelling and pain in the floor of the mouth was referred to the dentist. The patient was edentulous, and she has associated the lesion to the trauma of the complete dentures. The intraoral examination revealed a unique soft nodule, tender to palpation, covered with clinically normal mucosa, well-circumscribed, sessile, located at the floor of the mouth, in the anterior left region of the mandible, measuring 1.1 × 0.9 × 0.7 cm. Upon extraoral examination, there were no palpable lymph nodes. Her medical history was noncontributory. The clinical hypothesis was of inflammatory fibrous hyperplasia or mucus retention cyst. An excisional biopsy was performed, and the surgical specimen was well defined, with no attachment to surrounding tissues.

**Figure 1:**
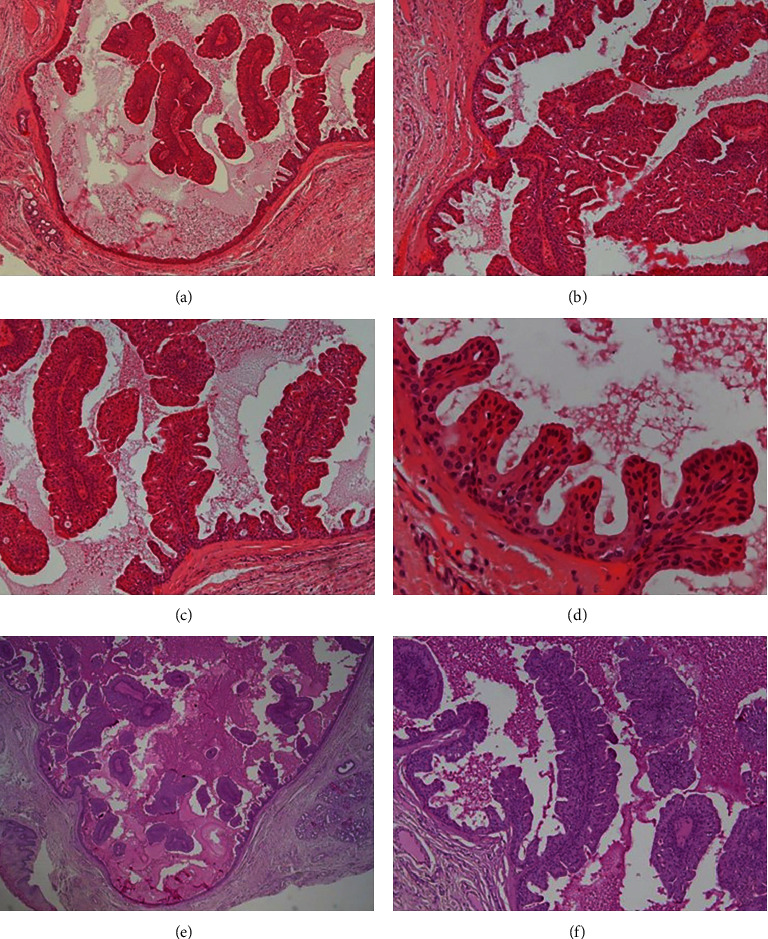
Histopathological section of the oral intraductal papilloma. (a) The cystic cavity filled by papillary tumor projections and anastomosing proliferations of columnar epithelial cells in a fibrovascular core (haematoxylin and eosin stain, H&E, original magnification ×5). (b–d) Note that the cystic wall is lined by uniform layers of cuboidal/columnar oncocytic epithelial cells, with no nuclear atypia or mitotic figures (H&E, original magnification ×10 (b), ×20 (c), ×40 (d)). (e, f) Note the mucous cells showing positivity for periodic acid-Schiff, presence of mucous material in the lumen and minor salivary gland acini in fibrous tissue (P.A.S., ×5 (e), ×20 (f)).

The histopathological analysis revealed a well-circumscribed unique cystic cavity arising from a minor salivary duct gland characterized by papillary projections of the cuboidal/columnar mucous and oncocytic cells to the cystically dilated ductal space and with no nuclear atypia or mitotic figures. The cystic lumen was partially filled by many branching papillary elements, consisting of thin strands of fibrovascular cores, surfaced by columnar cells and by a mucous fluid. The lesion is surrounded by a thick, fibrous tissue wall (Figures 1(a)–1(d)). Mucous secretory cells and mucous material exhibited positivity for periodic acid-Schiff staining (P.A.S.) (Figures 1(e) and 1(f)). Based on clinical and microscopical features, the final diagnosis was of an oral intraductal papilloma arising from a minor salivary gland obstructing the duct and mucus retention. The postoperative course was uneventful and no signs of recurrence during fourteen months of follow-up.

## 3. Discussion

Although rare, oral intraductal papillomas are benign solitary tumors arising from the excretory duct characterized by intracystic papillary growth and duct-like structures [1]. Clinically, patients with this lesion, as reported in our case, usually present small submucosal asymptomatic nodules, with less than 1.0 cm in diameter, variable duration, resembling a mucocele or inflammatory fibrous hyperplasia [1, 4, 5]. Most of these oral benign tumors are asymptomatic [1, 4], and in our patient, probably, the pain was associated with a chronic irritation in the lesion region due to the pressure of denture. Additionally, the occurrence of intraductal papilloma in a minor salivary gland in the present patient played a significant role in the obstruction of the salivary gland duct causing the mucus retention (Figures 1(a), 1(c), 1(e), and 1(f)), which clinically suggested a mucous retention cyst [5].

Microscopically, intraductal papillomas, as described in Figure 1, are usually well-circumscribed unilocular lesion exhibiting true intracystic papillary projections of ductal epithelium composed of small cuboidal cells arranged around a central lumen [2, 3, 5, 6]. The lumen is partially or completely filled by branching papillary elements consisting of a fibrovascular core, and the epithelium that lines the cystic-like cavity is the same type of epithelium of papillary fronds, causing the mucus retention [2–5, 7]. The presence of a varying degree of oncocytic metaplasia, as observed in the present case, is common in duct cysts arising from minor salivary glands [6].

The incidence of tumors that arise from the salivary ductal glands is difficult to determine because different terminology has been used for the denomination of the same lesion, and there have been less than 50 cases of oral intraductal papilloma in minor salivary glands [1]. In a review of salivary ductal papillomas, Brannon et al. [1] reported at least 40 intraductal papillomas that occurred in patients with 50 years old or older affecting predominantly the minor salivary glands of the lip and buccal mucosa. Additionally, the presence of intraductal papillomas in the minor salivary gland of the sublingual region is extremely rare, and there are only two reported cases in English literature [4, 5]. Therefore, the present case is the third intraductal papilloma report that developed in the sublingual region, involving a minor salivary gland. Although with good prognosis and low recurrence rates, it is important to distinguish intraductal papillomas of other ductal neoplasms of salivary ductal origin, as papillary cystadenoma, cystadenocarcinoma, sialadenoma papilliferum, and inverted ductal papilloma [1, 2, 6].

In contrast with intraductal papilloma, cystadenoma and cystadenocarcinoma show a multicystic well-circumscribed lesion lined by papillary epithelium with variable size [2, 6]. However, the cystadenocarcinoma is covered by a papillary epithelium with remarkable nuclear and structural atypia, some mitosis, and invasion of the surrounding tissue or infiltration of the adjacent salivary gland [6].

Additionally, sialadenoma papilliferum is characterized by a combination of exophytic projection of the surface squamous epithelium and inward papillary proliferation of duct epithelial cells [1, 2, 6, 7]. The underlying glandular component forms papillary extensions surfaced by columnar or cuboidal cells or both, which is contiguous with underlying arborizing ductal structures and cystic spaces. According to Nakaguro et al. [6], based on histological and genetic aspects, sialadenoma papilliferum may be considered to be a counterpart of syringocystadenoma papilliferum of the skin.

The distinction between intraductal and inverted papillomas residues in the epidermoid cell component, in which intraductal papilloma is a well-circumscribed unicystic lesion and inverted ductal papilloma expands to the surrounding connective tissue [1, 3, 6]. Also, inverted ductal papilloma demonstrates epithelial proliferation within the excretory duct leading to an opening of the lesion on to the mucosal surface, while intraductal papilloma is situated at a deeper level [1].

Concluding, our case reported reinforces that, although rare, the oral intraductal papilloma arising from the minor salivary gland may be an etiologic factor of mucus retention cyst in the mouth and the histopathological analysis is essential to establish the precise diagnosis.
